# Depiction of the In Vitro and Genomic Basis of Resistance to Hop and High Hydrostatic Pressure of *Lactiplantibacillus plantarum* Isolated from Spoiled Beer

**DOI:** 10.3390/genes14091710

**Published:** 2023-08-28

**Authors:** Joanna Bucka-Kolendo, Despoina Eugenia Kiousi, Adrian Wojtczak, Agapi I. Doulgeraki, Alex Galanis, Barbara Sokołowska

**Affiliations:** 1 Department of Microbiology, Prof. Waclaw Dabrowski Institute of Agricultural and Food Biotechnology, State Research Institute, Rakowiecka 36 Street, 02-532 Warsaw, Poland; adrian.wojtczak@ibprs.pl (A.W.); barbara.sokolowska@ibprs.pl (B.S.); 2 Department of Molecular Biology and Genetics, Faculty of Health Sciences, Democritus University of Thrace, 68100 Alexandroupolis, Greece; dkiousi@mbg.duth.gr (D.E.K.); agalanis@mbg.duth.gr (A.G.); 3 Laboratory of Food Microbiology and Hygiene, Department of Food Science & Technology, Faculty of Agriculture, Aristotle University of Thessaloniki, 54124 Thessaloniki, Greece; adoulgeraki@agro.auth.gr

**Keywords:** lactic acid bacteria, hop resistance, high hydrostatic pressure, whole genome sequencing, spoiled beer, *Lactiplantibacillus plantarum*, *Levitlactobacillus brevis*

## Abstract

Among the beer-spoiling microorganisms, the dominant ones belong to the genera *Lactobacillus*, *Leuconostoc*, *Oenococcus*, and *Pediococcus*. It is assumed that resistance to hop bitters correlates with resistance to other factors and can significantly impact the brewing industry. Beer preservation with high hydrostatic pressure eliminates the spoiling microorganisms while preserving all desired properties of the beer. Here, we present comprehensive in vitro and genomic analysis of the beer-spoiling *Lactiplantibacillus plantarum* KKP 3573 capacity to resist hop and high hydrostatic pressure. *Lp. plantarum* KKP 3573 is a strain isolated from spoiled beer. Our finding suggests that the growth rate of the strain depends on the medium variant, where a small concentration of beer (5 IBU) stimulates the growth, suggesting that the limited concentration has a positive effect on cell growth. At the same time, increased concentrations of 20 IBU, 30 IBU, and pure beer 43.6 IBU decreased the growth rate of the KKP 3573 strain. We observed that higher extract content in the pressurized beer increased microbial survivability. The wort and Vienna Lager beer can stimulate the baroprotective effect. The taxonomy of the novel strain was confirmed after whole genome sequencing (WGS) and comparative genomic analysis. More specifically, it contains a chromosome of 3.3 Mb with a GC content of 44.4%, indicative of the *Lp. plantarum* species. Accordingly, it possesses high genomic similarity (>98%) with other species members. Annotation algorithms revealed that the strain carries several genes involved in resistance to stress, including extreme temperature, hop bitters and high pressure, and adaptation to the brewing environment. Lastly, the strain does not code for toxins and virulence proteins and cannot produce biogenic amines.

## 1. Introduction

Beer is the most widely consumed alcoholic beverage in the world. The concentration of alcohol (ranging from 0.5 to 10%), bitter hop acids (estimated range of iso-α-acids from 17 to 55 ppm), the presence of 0.5% CO_2_, sulfur dioxide, and also dissolved oxygen deficiency (<0.3 ppm) suggests that beer is a microbiologically stable beverage [1,2,3]. It is also poor in nutrients as the fermentative activity of brewer’s yeast almost depletes them [1,2,4]. The microbial stability is achieved by using heat treatment, which often leads to quality deterioration. However, high-pressure processing (HPP) effectively inactivates vegetative microorganisms without the influence of thermal treatment [5]. HPP can be used for liquid products and solids with high moisture content. Studies have shown that the main advantage of applying high hydrostatic pressure (HHP) is inactivation of undesirable microorganisms, improvement in microbiological safety, and preservation of the organoleptic properties of beer and wine, which can extend shelf life [5,6,7,8,9]. Since beer production requires high starch content in barley malt due to the mashing process in which the starch is saccharified to produce fermented sugar, it turned out that pressure can effectively improve enzymatic saccharification during the malting process under appropriate conditions [10]. HHP treatment did not affect wheat beer’s main quality characteristics, including original extract, ethanol content, pH, and bitterness, and increased the beer’s foaming and haze characteristics [11]. Therefore, HHP may be a promising nonthermal method for wheat beer production without affecting the original characteristics [12]. 

The presence of microorganisms in beer, which causes spoilage, adversely affects the sensory properties of this beverage. Some microorganisms tolerate beer parameters and lead to changes in beers, like turbidity, sedimentation, acidity, sometimes with a diacetyl flavor [1,2], and unpleasant odor caused by compounds such as butyric acid, caproic acid, and hydrogen sulfide [13]. In a recent review, the methods for detecting and identifying beer-spoiling microorganisms were summarized by Oldham and Held, 2023 [14]. Beer spoilage microorganisms range from Gram-positive and Gram-negative bacteria to fungi, including wild yeasts and molds. The presence of LAB in breweries can be harmful, where *Lactobacillus* and *Pediococcus* are the most common contaminants in beer, accounting for 60–90% of all spoiling [2,15]. 

LAB shows different beer spoilage capacities, and the response of individual strains to hop compounds differs [16]. Some *Lactobacillus* can grow extensively and spoil almost any type of beer [2], while others do not. This genus is very diverse, and some members exhibit intrinsic tolerances and stress responses that allow them to survive in a harsh environment like beer [5,17]. Moreover, the spoilage potential of *Lactobacillus* must be determined in a short time to take preventive measures against this contamination. How quickly spoilage occurs depends on time and temperature. To develop in such difficult conditions, bacteria have to develop adaptive mechanisms [3], as intraspecific differences in hop tolerance cannot be predicted by differences in cell or colony morphology, growth pH, carbohydrate metabolism, manganese requirements, superoxide sensitivity, or cellular protein expression [6,18,19]. This could be attributed primarily to their acquired ability to grow in the presence of hops. Studies show that hop compounds cause membrane damage, a decrease in intracellular pH, and a reduction in the size and number of *Lp. plantarum* [6,20] or *Lv. brevis* cells [5,21]. In addition, only a small subpopulation within hop-tolerant strains retains membrane integrity when exposed to hops at low pH. These cells have been shown to act as ionophores of a mobile carrier, which causes a decrease in intracellular pH and an increase in the concentration of divalent cations, particularly Mn_2_C [22], and contributes to growth. In addition, a large amount of Mn_2_C increased the viability of cells on hops [21]. The cell wall of LABs spoiling beer exhibits galactosylation of glycerol teichoic acid, which hinders the penetration of hop acids into the cell. The amount of lipoteichoic acid in the bacterial cell wall is higher in beer-spoiling strains. In these strains, ATP and ATPase activity increases [17,19]. Microscopic observations indicate that the LABs in beer are shaped like shorter sticks, suggesting that the smaller cell surface area benefits defense mechanisms [1,2]. 

**Figure 1:**
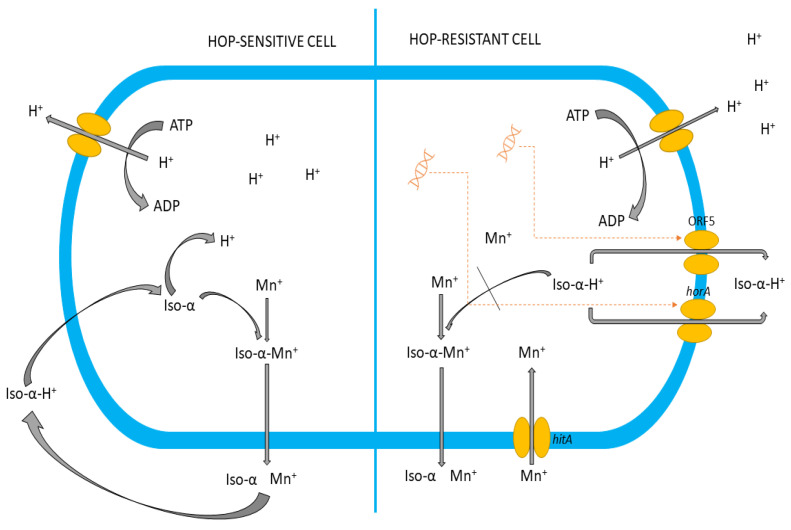
Schematic overview of hop-related mechanisms in Gram-positive bacteria [27].

The microbial response to stress conditions like the hop compound was found to be explained using tools like single-cell analysis [21]. Figure 1 illustrates the hop-related mechanisms of bacterial inhibition in beer. Research is being undertaken to determine which genes in *lactobacillus* are responsible for the bacteria’s ability to spoil beer [5,23,24,25]. Detection of marker sequences is essential for better risk assessment in the brewing industry [19]. Beer spoilage could be mainly correlated to the genes responsible for tolerance to hop compound [13,15] cases [17,26]. 

In lactobacilli, hop resistance genes have been identified on plasmids *horA*, *horC*, and *hitA* [2,13,28,29,30], but their presence or expression does not always correlate with the ability of LABs to grow in beer [31]. The *hitA*, *horA*, and *horC* genes are not found in a consistent combination in beer spoilage bacteria [2,13,17,32,33]. Most likely, other as of yet uncharacterized products of genes are present on specific plasmids responsible for beer spoilage. These novel gene products may function well with plasmid-encoded HorA, HorC, and HitA [26]. In *Lp. plantarum* and *Lv. brevis* resistance to hop compounds, HorA activity and ATP-binding multidrug resistance transporter (ABC), conferring resistance to hop compounds, were detected [6,32].

The potential risk of the presence of adapted cells to stress is crucial during beer processing. The physiology of strains that survive HHP simulates one of the resistant cells under other various stresses [34]. Currently, research is focused on analyzing the response of microorganisms to HHP-induced stress by assessing its impact on the structure, metabolism, growth, and viability of cells [5,35]. Under the influence of HHP, the cell membrane’s fluidity decreases, leading to a decrease in transmembrane transport and loss of flagellum motility. The membrane is usually the first cell elements to be damaged by high pressure [36]. Other studies show that high pressure inhibits the synthesis of ATP in microorganisms and can also activate or deactivate the enzyme, denature functional proteins, and lead to a reduction in proton flow, reducing intracellular pH [34]. Specific gene regulation for stress resistance mechanisms involves accumulating significant amounts of heat shock proteins (HSPs) in the cell [37]. Transfer or elimination of regulatory genes related to pressure resistance affects the pressure tolerance of a strain [38]. The stress response HPP uses subsets of other responses rather than evoking a specific reaction to HPP. As a part of the cross-regulation mechanism in HHP, the expression of genes regulated with regulons CtsR and HrcA were analyzed [23]. Studies have shown that the relative amount of mRNA of many genes involved in the stress mechanism can result from selective transcription or mRNA stability under HHP.

This study investigated the tolerance to different hop concentrations and the response to HHP treatment of the beer-spoiling strain KKP 3573 in vitro. Furthermore, whole-genome sequencing and annotation were performed to determine the strain’s phylogenomic and genomic characteristics, focusing on annotated genes involved in spoiling and viability during stresses, including HHP.

## 2. Materials and Methods

### 2.1. Strain and Growth Conditions and Molecular Identification

The strain was isolated from spoiled beer and grown on MRS agar (DeMan, Rogosa, and Sharpe, Merck KGaA, Darmstadt, Germany) and UBA medium (Universal Beer Agar, Merck KGaA) at 30 °C for 72 h under anaerobic conditions. The strain was identified with the MALDI-TOF MS system and *16S* rDNA analysis, as Bucka-Kolendo et al., 2020 described previously [39]. The strain under number KKP 3573 was deposited in the Culture Collection of Industrial Microorganisms—Microbiological Resource Center (IAFB, Warsaw, Poland). The *16S* rDNA sequence of strain was deposited in the GenBank NCBI database under the accession number OK287291. 

### 2.2. Hop Resistance of KKP 3573 Strain

To determine the resistance to hop, the growth kinetics of the KKP 3573 strain was estimated with the automated growth curve analysis system Bioscreen C Pro (Oy AB Ltd., Growth Curves, Finland), as described by Kiousi et al., 2023 [25]. London Ale beer was used to formulate the starting concentration of 40 IBU, from which other IBU concentrations—5, 10, 20, and 30—were prepared to perform hop resistance analysis on the Bioscreen. The London Ale contained 5.79% alcohol (*v*/*v*) and 43.6 IBU (International Bitterness Units) and a mix of different hop compounds, mainly α-acids, iso-α-acids, xanthohumol, and iso-xanthohumol. All beer analyses were performed using the European Brewery Convention (EBC) and Mitteleuropäische Brautechnische Analysenkommission (MEBAK) methods. Preceding the experiment, 18 h cultivation was conducted. Subsequently, the culture was adjusted to an OD of 0.5. After this adjustment, 50 µL of 0.5 McF microbial culture (corresponding to 10^7^ CFU/mL) was inoculated in MRS broth (Merck KGaA, Darmstadt, Germany) and applied to wells with 250 µL medium (Table 1), the research was performed for 72 h at 30 °C, and the OD_600_ was registered every hour in triplicates. Non-inoculated MRS broth and all media containing various hop concentrations were used as negative control.

The scheme of applying the bitterness concentration to evaluate the hop resistance for the studied strain is presented in Table 1. All components of the media were sterilized using filtration for the beer and autoclaved at 121 °C for 15 min for the remaining nutrient ingredients. The sterile components were then mixed under aseptic conditions.

After determination of the growth curve, a Gompertz curve was fitted to the data using the LabPlot 2.9.0 program (KDE).

$$L_{t}=A+C\times e^{{-e}^{-B\times \left(t-D\right)}}$$

where *L_t_*—OD at time *t*; *t*—time (h); *A*—asymptotic OD value as *t* decreases indefinitely; *B*—relative growth rate at *D*; *C*—the asymptotic amount of growth that occurs as *t* increases indefinitely; *D*—time at which the absolute growth rate is at its maximum (h).

The maximum growth rate *μ_max_* was determined based on the Gompertz model.

$$\mu_{max}=\frac{B\times C}{e}\left[h^{-1}\right]$$

The change in optical density (Δ*OD*) was determined based on the difference between *OD_max_* and *OD_min_*.

$$\Delta OD={OD}_{max}-{OD}_{min}$$

where *OD_max_*—the highest value of optical density observed during the process; *OD_min_*—the lowest value of optical density observed during the process.

Statistical analyses were performed using a one-way variance analysis (ANOVA) with Tukey’s HSD test (α = 0.05) using Statistica 14.0 (TIBCO Software, Palo Alto, CA, USA). The presented data are a mean ± standard deviation (SD), with the normality distribution checked using the Shapiro–Wilk test. 

### 2.3. HHP Application

Two types of beer and one wort were used in the pressurization process: The Vienna Lager type, unfiltered with 5.9% alcohol (*v*/*v*), with 14.2°Blg (Balling degrees), and the Pale Lager type, with 4.8% alcohol (*v*/*v*) and 10.1°Blg (Table 2). The wort was prepared as an aqueous solution of Merck’s Malt extract broth, with pH 4.8 ± 0.2. 

The strain exposed to HHP was in the early stationary phase. The cells were harvested using centrifugation at 4 °C for 10 min at 4000× *g* from the cultures in MRS broth. After washing in phosphate buffered saline (PBS) (pH 7.2) three times, they were inoculated in samples at 8 log (CFU/mL). Then, samples were dispensed in 4 mL portions in sterile plastic cryovials (Simport, Saint-Mathieu-de-Beloeil, QC, Canada). The process was performed using U 4000/65 apparatus (Unipress, Warsaw, Poland) with a treatment chamber of 0.95 L vol and a maximum working pressure of 600 MPa. Distilled water and polypropylene glycol (1:1, *v*/*v*) were used as a pressure-transmitting medium. The samples were subjected to a pressure of 300 MPa, 400 MPa, and 500 MPa for 5 min; the pressurization times reported did not include the come-up and come-down times. The process was carried out at room temperature. The temperature was measured in the chamber, and the increase during pressurization was 6 °C/500 MPa. Each pressure process was performed for two parallel samples.

The statistical analyses were performed using a multiway variance analysis (MANOVA) with Tukey’s HSD test (α = 0.05). The presented data are a mean ± standard deviation (SD), with the normality distribution checked using the Shapiro–Wilk test.

This was followed by a Spearman’s rank correlation test to examine the relationship between survivability and the alcohol and extract content.

All the analyses were performed using Statistica 14.0 (TIBCO Software, Palo Alto, CA, USA).

### 2.4. Determination of the Number of Surviving Cells in the Pressurization Process

The number of populations surviving the pressure processes was determined using the pour plate method in MRS agar medium according to ISO 15214:2002 [40] standard. The plates were incubated for 72 ± 3 h at 30 °C. After the incubation period, colonies were counted on plates from two successive dilutions containing not less than 10 and not more than 300 colonies. Determination of the number of lactic acid bacteria means determining the number of colony-forming units (CFU) per milliliter sample.

The number of bacteria (*L*) in 1 mL was calculated according to the following formula:

L=C×d/(N1+0.1N2)

where *C*—the sum of colonies on all plates selected for counting; *N*1—number of plates from the first counted dilution; *N*2—number of plates from the second counted dilution; *d*—dilution factor corresponding to the first (lowest) calculated dilution.

### 2.5. MALDI-TOF MS Analysis

The MALDI-TOF MS technique was used to identify the strain with the MALDI-Biotyper 3.0 software (Bruker Daltonik, Bremen, Germany), and to analyze and compare the unpressurized and pressurized KKP 3573 strain mass spectra with 5291 reference spectra. The α-cyano-4-hydroxycinnamic acid (HCCA) matrix solution was used due to its better sensitivity, higher intensity, and higher number of signals in the lower mass range. Identification of the strain was based on the criteria proposed by the manufacturer, where a score higher than 2.30 and in-between 2.30 and 3.00 indicates the identification result as highly probable at the species level; a score between 2.00 and 2.29 indicates probable identification at the species level; a score between 1.70 and 1.99 assigns identification to the genus level; and a score below 1.70 is not reliable for identification. The analysis was performed according to Bucka-Kolendo et al., 2020 [41].

The visualization of the compared mass spectra profiles (MSP) for the KKP 3573 strain was carried out using the mMass—Open Source Mass Spectrometry Tool (http://www.mmass.org/, accessed on 5 July 2023). The mass spectra for the unpressurized strain KKP 3573 and the strain pressurized at 300 MPa/5 min were compared.

### 2.6. Genome Sequencing

Genomic DNA from the KKP 3573 strain was isolated using DNeasy PowerFood Microbial Kit (Qiagen, GmbH, Hilden, Germany) according to the manufacturer’s protocol and as described by Kiousi et al., 2023 [25]. Briefly, DNA purity was determined with Nanodrop ND-1000 Spectrophotometer (Thermo Fisher Scientific, Watertown, MA, USA), and DNA concentration was measured with Qubit 4.0 Fluorometer (Qubit dsDNA BR Assay Kit, Invitrogen, Carlsbad, CA, USA). The genomic DNA library was prepared with the Illumina DNA Prep kit (Illumina, San Diego, CA, USA) according to the manufacturer’s instructions (number #1000000025416v09), and a manual normalization step was performed based on library concentration and average size. Sequencing analysis of genomic DNA was performed with an Illumina MiSeq sequencing platform using 2 × 151 bp paired-end MiSeq protocol and reagent v3 (600-cycle) kit. 

A total of 1,505,060 paired-end reads were obtained for *Lp. plantarum* KKP 3573. FASTQC (v0.11.9) [42] was used to determine the quality of the obtained reads and Trimmomatic was utilized to discard low-quality sequences (version 0.39) [43]. The genome was assembled de novo using a previously published pipeline [19] using SPAdes, plasmidSPAdes (version 3.15.1) [44] to extract plasmid sequences and SSPACE for genome scaffolding [45]. The Quality Assessment Tool (QUAST, version 5.2.0) was utilized to calculate assembly metrics and genome quality [46]. The genome map was constructed using the Proksee server [47]. 

### 2.7. Phylogenomic Analysis

The genomic sequences (chromosome level of assembly) of 39 *Lp. plantarum* strains were obtained from the NCBI Assembly database using a python script. Average Nucleotide Identity (ANI) was calculated with Pyani (version 0.2.10) [48] for taxonomic purposes. Whole-genome sequences of closely and distantly related lactobacilli and *Staphylococcus aureus* strain NCTC 8325 were aligned using progressiveMauve [49]. “Interactive Tree of Life” (iTol; version 6.1.1; [50]) was employed for the visualization of the resulting phylogenomic tree. 

### 2.8. Genome Annotation

Prokka (version 1.14.5) [51] and the local version of the Prokaryotic Genome Annotation Pipeline (PGAP) [52] were used for genome annotation. Online tools were used to annotate specific genetic elements; PlasmidFinder was used for the detection of plasmid sequences in the WGS of the strain [53], ISFinder (e-value cut-off: 0.01) [54] for insertion sequence elements, PHAge Search Tool Enhanced Release (PHASTER) [55] for prophage regions, CRISPRDetect (version 2.4; [56]) and CRISPR/Cas Finder (version 1.1.0) [57] for Clustered Regularly Interspaced Short Palindromic Repeats (CRISPR) arrays, Resistance Gene Identifier (RGI; version 5.2.0) [58] for the annotation of antimicrobial resistance genes, and VirulenceFinder 2.0 [24] to pinpoint genes involved in the manifestation of the virulence phenotype. The tools CARD-RGI (version 1.2.0) [58], mobileOG-db (version 1.1.2) [59] and Alien Hunter (version 1.1.0) [60] were utilized to annotate genes involved in antimicrobial resistance, mobile elements, and horizontal gene transfer regions in the genome map produced using Proksee. Bacteriocin clusters were annotated with BAGEL4 [61]. EggNOG-mapper (version 2.1.9) [62] and BlastKOALA (version 3) [63] were used to assign predicted proteins into Clusters of Orthologous Groups (COGs) and KEGG Orthology (KO) groups. Finally, the annotated genome of strain *Lp. plantarum* KKP 3573 was manually searched for genes involved in stress resistance (resistance to low pH, osmotic and oxidative stress) and hop tolerance. 

## 3. Results and Discussion

### 3.1. Beer-Spoiling Ability of Lp. plantarum KKP 3573 

This study aimed to investigate the growth rate of the KKP 3573 strain in different media variants containing varying concentrations of beer and iso-α acids (IBU) (Table 3). The growth rate was monitored by measuring the increased optical density (Δ*OD*) of the cultures over time.

**Figure 2:**
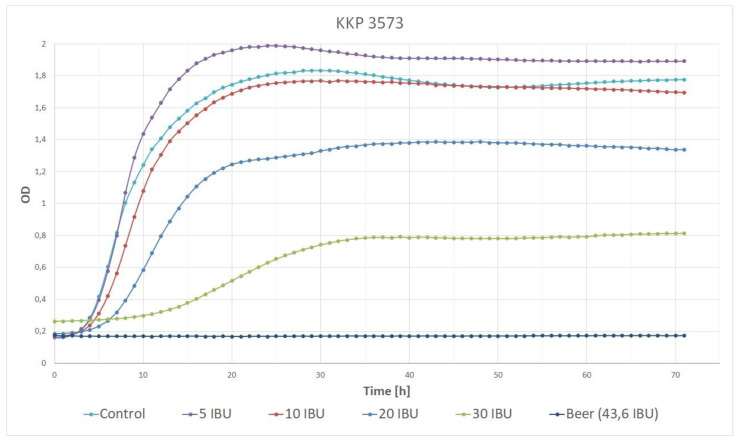
Growth curves of the strain *Lp. plantarum* KKP 3573 in MRS media containing various hop concentrations.

The findings indicated that the growth rate of strain KKP 3573 was dependent on the medium variant, with a decrease in the growth rate observed as the concentration of IBU increased (Table 3, Figure 2). This pattern was consistent across most media variants with the 30 IBU and beer with 43.6 IBU variants showing significant differences from the control. However, the 5 IBU variant was an exception to this trend, as the growth rate was significantly higher for strain KKP 3573 in this variant than the control (MRS broth).

This study also revealed that increasing the concentration of beer led to a decline in the growth rate and the number of cells, indicating a negative impact of hop, alcohol, and/or other beer ingredients on cell growth. This effect was observed in the 20 IBU, 30 IBU, and pure beer variants, where significantly decreased Δ*OD* values were counted.

Adding a small amount of beer with a concentration of 5 IBU stimulated growth for strain KKP 3573, suggesting a positive effect of low beer concentration on cell growth.

### 3.2. Influence of HHP on Lp. plantarum Strain Survivability

In 10% wort, the decrease in cell number (CFU/mL) after applying the pressure of 300 MPa for 5 min was 1.62 log for *Lp. plantarum* KKP 3573. The pressure of 400 MPa/5 min resulted in a significant cell inactivation in the range of 6.38 log and, at the same time, increasing the pressure to 500 MPa caused total inhibition of the strain.

In the Vienna Lager beer, after applying 300 MPa for 5 min, the cell number decreased, with a higher number for the *Lp. plantarum* strain KKP 3573 of 4.51 log (CFU/mL) being observed; applying 400 MPa and 500 MPa resulted in total inhibition. In Pale Lager beer, for the KKP 3573 strain, total inhibition was observed for all three pressures (300 MPa, 400 MPa, 500 MPa). 

**Figure 3:**
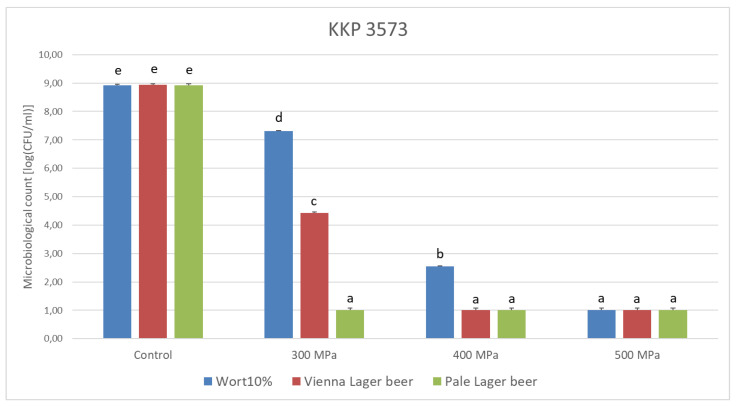
Comparison of media at different pressure levels. Lowercase letters indicate statistically significant differences between variants under different media and pressures.

The inactivation of *Lp. plantarum* cells in the pressure process was influenced by the strain type and the medium (Figure 3). The highest inactivation was found in Pale Lager beer, which indicates that this type of beer can be relatively easily preserved with HHP. During high-pressure treatment, the baroprotective effect of wort and compounds in unfiltered Vienna Lager beer on *Lp. plantarum* cells was observed.

The Spearman Rank Order Correlations revealed interesting findings for pressures up to 400 MPa. The analysis showed that the alcohol content is not significantly correlated with the quantity of microorganisms present. However, there is a significant correlation between the extract content and the number of microorganisms.

Furthermore, the analysis indicated that a higher extract content was positively correlated with increased microbial survivability. This finding suggests that a higher extract content in the samples was associated with a higher microbial survival rate under the specified pressure conditions. This observation could indicate that the nutrients or other factors present in the extract may have provided a more favorable environment for the microorganisms, allowing them to thrive and survive better. The premise of these results is consistent with the relative gene expression results shown by Bucka-Kolendo et al., 2021 [24].

### 3.3. The Impact of HHP on MALDI-TOF MS Identification

Strains after growth in optimal conditions (unpressured) and after being subjected to a pressure of 300 MPa for 5 min (pressurized) were analyzed with MALDI-TOF MS. The obtained mass spectra profiles (MSP) were investigated (Figure 3), and attained identification was compared with previously obtained phylogenetic affiliation.

The MALDI-TOF MS analysis identified the unpressured strain as an *Lv. brevis* species, with an average score of 2.33. According to the manufacturer Brucker, scores in the 2.30–3.00 range indicate a high probability of species identification. The result was confirmed with *16S* rDNA gene sequence analysis, identifying the strain as an *Lv. brevis* species with 99.88% gene sequence similarity [39]. The pressurized strain was identified as *Lp. plantarum*, with a score of 2.21, reflecting probable identification at the species level according to the producer (2.00–2.29). Additionally, identification based on the housekeeping gene phenylalanyl-tRNA synthase α subunit (*pheS*) sequence was performed. This method identified the strain as *Lp. plantarum* with 99.23% similarity. 

**Figure 4:**
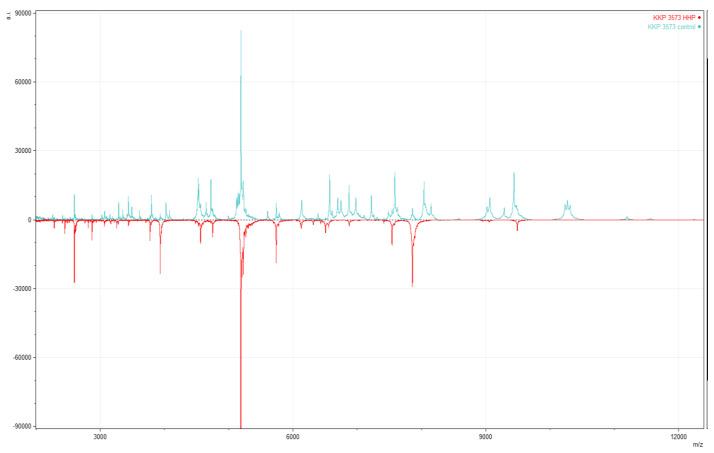
Changes in the mass spectra of *Lp. plantarum* KKP 3573. Unpressured strain—blue (upper) spectrum; pressurized strain—red (lower) spectrum.

Based on the MALDI-TOF MS analysis, a stress factor, such as HHP, can impact the changes in the protein profile. As is visible in Figure 4, when the mass spectra profiles for the unpressurized strain KKP 3573 and the strain treated with 300 MPa/5 min were compared, the different protein profiles from their protein mass fingerprinting analyses showed changes between the unpressured and pressured strain. Different protein profiles correlated with different identification for this strain when performed with MALDI-TOF MS. 

This misinterpretation led to performing whole-genome sequencing (WGS) to obtain the reliable and validated result of the strain phylogenetical affiliation.

### 3.4. Whole-Genome Sequencing, Gene Annotation, and Phylogenomic Analysis of Strain Lp. plantarum KKP 3573

**Figure 5:**
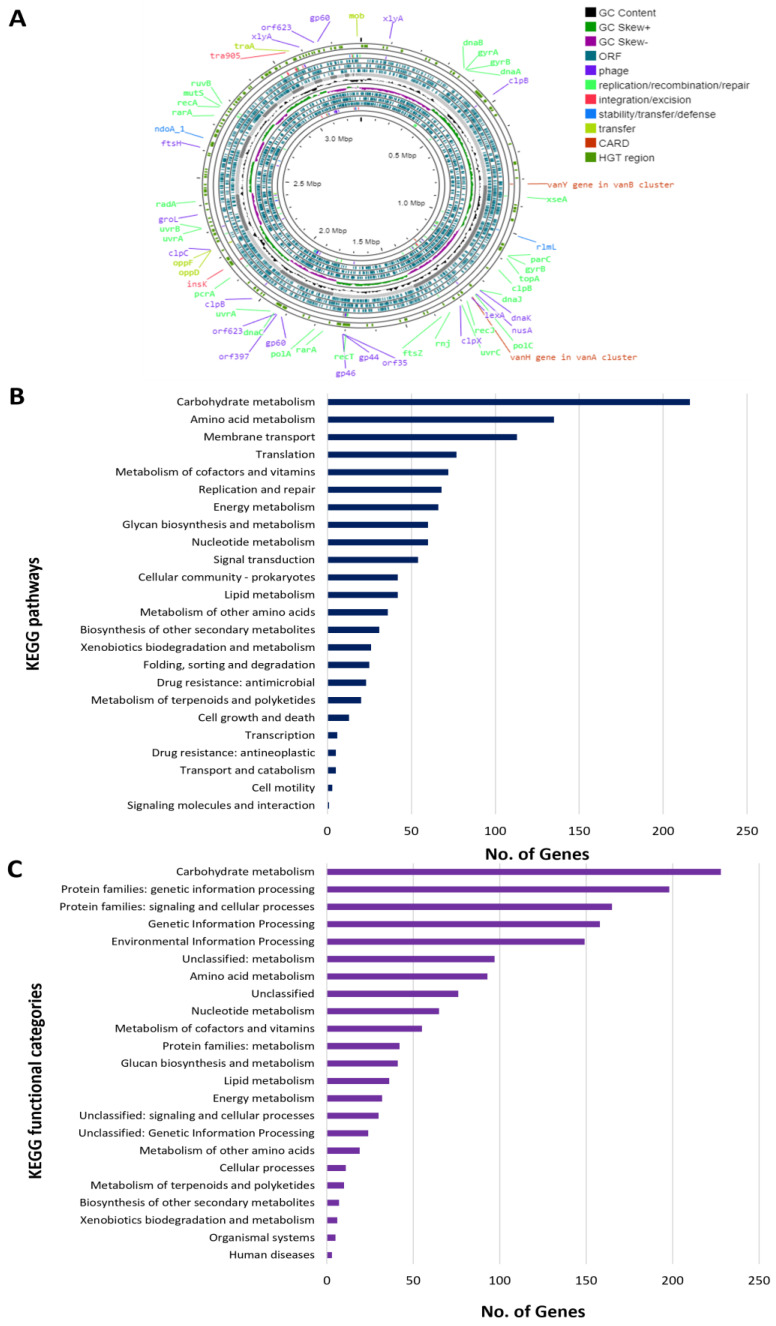
Whole-genome sequencing and assembly of *Lp. plantarum* KKP 3573. (**A**) Chromosome map of strain *Lp. plantarum* KKP 3573. Classification of proteins encoded by the strain into (**B**) KEGG pathways and (**C**) KEGG functional categories.

Whole-genome sequencing and assembly were performed to determine the genetic identity of the strain of interest. The chromosome of KKP 3573 has a length of 3.29 Mbp and GC content of 44.39% (Figure 5, Table 4), indicative of its classification as an *Lp. plantarum* strain. The WGS of the strain also contains two plasmids (repUS64 and rep28), as revealed using plasmidSPADES and PlasmidFinder 2.0 (Table S1). The *Lp. plantarum* KKP 3573 genome contains 39 insertion elements and three intact prophage regions (Table 5), while extensive regions in the genome resulted from horizontal gene transfer (Figure 5). Moreover, the strain does not contain CRISPR arrays or CAS proteins. Finally, the strain does not code for virulence factors or transferable antibiotic resistance genes. 

The annotated genes of the strain were further categorized into 19 clusters of orthologous groups using EggNOG. The two most represented groups are carbohydrate metabolism and transport (E) and transcription (K), followed by amino acid metabolism and transport (E) (Table 5). Of note, the majority of genes possess unknown functions (19%). Furthermore, the most represented COG category in the *Lp. plantarum* pangenome is replication and repair (L), followed by groups G and K. Furthermore, predicted proteins were assigned to 206 KEGG pathways, organized into 24 functional categories (Figure 5). Most proteins are assigned to the “carbohydrate metabolism” category (216 proteins), followed by the “amino acid metabolism” (135 proteins) and “membrane transport” (113 proteins) functional categories. Concerning the KEGG pathway assignment, most annotated proteins are involved in “carbohydrate metabolism” (228 proteins) or “genetic information processing” (198 proteins).

**Figure 6:**
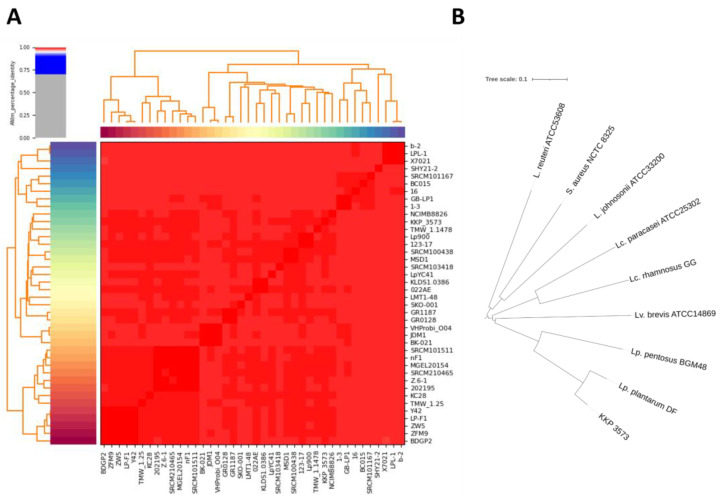
Phylogenetic analysis of the novel strain *Lp. plantarum* KKP 3573. (**A**) ANI of strain *Lp. plantarum* KKP 3573 with members of the *Lp. plantarum* species. (**B**) Phylogenomic tree based on the WGS of selected type strains belonging to the *Lp. plantarum* or other closely or distantly related species. *S. aureus* NCTC 8325 was used as an outgroup. The tree was constructed on the iTOL server.

The ANI and phylogenetic relationships with other lactobacilli were determined using established algorithms to validate the phylogeny of the novel strain. Strain *Lp. plantarum* KKP 3573 presents high genomic similarity with other species members (>98.9%), validating its classification in the *Lp. plantarum* species. Accordingly, phylogenetic analysis based on the WGS of the strain showed that it clusters with other members of the species (Figure 6).

### 3.5. Lp. plantarum KKP 3573 Possesses Genes Involved in Tolerance to Stress and the Beer-Spoiling Phenotype

Several genes involved in the strain’s capacity to persist in environmental stress conditions were annotated in the WGS, as shown in Table 6. More specifically, *Lp. plantarum* KKP 3573 possesses the *atpABCDEFGH* cluster coding for a F0-F1 ATPase and the gene *yvgP* coding for a sodium–proton antiporter, conferring tolerance to low pH. Furthermore, several proteins involved in heat and cold shock resistance were annotated in the genome of the novel strain. These genes may belong to the HSP20 family or are multichaperone systems that ensure cellular integrity and recovery after exposure to extreme temperatures. The strain also carries genomic features indicative of osmotic shock tolerance, including *grpE* and the *opuABCD* cluster. Accordingly, genes involved in oxidative stress response and oxygen tolerance (*nox*, *gpo*, and *tpx*) were annotated in the WGS of *Lp. plantarum* KKP 3573. High-pressure resistance is conferred via a multitude of mechanisms, correlated with high transcription or activity levels of proteins involved in heat shock response and SOS response triggered by environmental stresses that result in DNA damage [64,65]. In this context, *Lp. plantarum* KKP 3573 contains the machinery that could be used to ensure viability during HHP, including *dnaK* and *lon*. Additionally, the strain contains genes for *ctsR* and *hrcA*, two transcriptional regulators, that were shown to be involved in HHP response.

The beer matrix is a hostile niche for bacterial growth due to the presence of hop bitters. In this context, multiple genes involved in the export of bitters were annotated. More specifically, the strain carries three copies of the *mntH* gene coding for an H(+)-stimulated, divalent metal cation uptake system that regulates the detoxification of hop bitters. Additionally, a full cluster for unsaturated fatty acids biosynthesis was identified. Gene *fabZ* is thought to be involved in the capacity of strains to withstand the beer microenvironment. All genes involved in hop resistance are chromosomally encoded.

The beer-spoiling phenotype can be attributed to several phenotypic properties of bacteria. EPS production and biofilm formation mainly contribute to the phenotypic changes related to beer spoilage. In this context, genes involved in EPS production (*epsB*) and biofilm formation (*luxS*) were annotated in the chromosome of the strain. Finally, the capacity of strains to produce antimicrobial metabolites could negatively affect matrix microbiota, ultimately influencing the organoleptic characteristics of fermented beverages. The use of BAGEL4 and consecutive comparative genomic analyses resulted in the identification of two plantaricin clusters. More specifically, the *Lp. plantarum* KKP 3573 strain carries complete clusters for the production of the two-peptide, class II plantaricins EF and JK (Figure S1). The mature core peptides of the clusters present 100% sequence identity and structural conservation with other family members and with functionally characterized peptides produced by *Lp. plantarum* C11 (Table S2). Additionally, biosynthetic pathways for the production of the small antimicrobial molecules were identified. *Lp. plantarum* KKP 3573 carries the biosynthetic machinery for secretion of L-lactate (FMN-dependent L-lactate dehydrogenase) and of hydrogen peroxide (NADH oxidases, multicopper oxidase) (Table 6).

Next, we sought to determine the possible detrimental effects of strain consumption on the host’s health. The strain *Lp. plantarum* KKP 3573 does not contain virulence genes or genes involved in the production of hemolysins. Accordingly, it does not carry transferable antimicrobial resistance genes. However, the strain may be resistant to vancomycin, as it carries chromosomally encoded *vanH* and *vanY* genes. Furthermore, the capacity of the strain to code for biogenic amines was examined in silico. Biogenic amines are derived from the catabolism of proteins [66]. Enzymes involved in the formation of biogenic amines are amino acid deiminases and decarboxylases. *Lp. plantarum* KKP 3573 does not code for these enzymes, and it therefore may not be able to produce these detrimental compounds in situ.

## 4. Conclusions

In this study, we described the capacity of a novel *Lp. plantarum* strain isolated from spoiled beer to present resistance to hop and high hydrostatic pressure in vitro and in silico. 

We demonstrated that a small concentration of hop can stimulate the growth of the *Lp. plantarum* KKP 3573 strain when increased concentration can affect decreasing the microbial growth. The annotation algorithms revealed that the strain carries several genes involved in the stress resistance mechanism, such as temperature, hop bitterness, and high pressure. As a consequence of the strain pressurization, a baroprotective effect may occur. This knowledge is important for a better understanding of the conditions that may favor the growth of this microorganism in beer and, subsequently, the actions that we have to take in order to avoid its growth in a real environment. 

In brief, understanding the relationship between hop content and microbial survivability is crucial, as it sheds light on the potential impact of specific components within the beer on microbial performance under pressure. It also emphasizes the importance of considering extract content as a relevant factor when assessing microbiological characteristics in the context of high-pressure conditions. Further research exploring the underlying mechanisms behind this correlation could provide valuable insights into optimizing food preservation techniques and microbial safety in high-pressure processing applications. 

## Figures and Tables

**Table 1:** Scheme of prepared different bitterness units (IBU) for the Bioscreen analysis.

	Control	5 IBU	10 IBU	20 IBU	30 IBU	Beer 43.6 IBU
MRS broth concentrate (2×)	50%	50%	50%	50%	-	-
MRS broth concentrate (4×)	-	-	-	-	25%	-
Water	50%	12.5%	25%	-		-
Beer (40 IBU)	-	37.5%	25%	50%	75%	-
Beer (43.6 IBU)	-	-	-	-	-	100%

**Table 2:** Comparison of parameters for Vienna Lager and Pale Lager beers.

	Vienna Lager Beer	Pale Lager Beer
Alcohol, % (m/m)	4.62 ± 0.16	4.31 ± 0.15
Alcohol, % (*v*/*v*)	5.91 ± 0.16	5.45 ± 0.15
Apparent Extract, % (*w*/*w*)	2.88 ± 0.09	<0.50
Real Extract, % (*w*/*w*)	4.99 ± 0.06	2.03 ± 0.03
Original Wort Extract, % (m/m)	13.85 ± 0.14	10.46 ± 0.11
Bitterness (International Bitterness Units—IBU)	20.0	20.4

**Table 3:** Results for growth rate coefficients (*μ*) and optical density difference (Δ*OD*) for KKP 3573 strain.

Medium	KKP 3573	
	*μmax*	Δ*OD*
Control (MRS)	0.170 ± 0.005 e	1.675 ± 0.039 e
5 IBU	0.170 ± 0.005 aE	1.675 ± 0.039 aE
10 IBU	0.235 ± 0.005 aF	1.828 ± 0.013 aF
20 IBU	0.163 ± 0.007 aD	1.602 ± 0.034 aD
30 IBU	0.099 ± 0.005 aC	1.204 ± 0.018 aC
Beer 43.6 IBU	0.031 ± 0.001 aB	0.534 ± 0.026 aB ^1^

^1^ Lowercase letters indicate statistically significant differences for *μ_max_* and Δ*OD* between medium variants. Uppercase letters indicate statistically significant differences for *μ_max_* and Δ*OD* between medium variants for the same strain.

**Table 4:** Genome characteristics of *Lp. plantarum* KKP 3573.

Genome Characteristics	Value
Length	3,295,227 bp
GC content	44.39%
Total genes	3.102
CDSs	3.052
rRNAs	4
tRNAs	42
ncRNAs	4
Pseudogenes	29
No. of CRISPR arrays	0
IS elements	39
Phages:	
Intact	3
Incomplete	3
Questionable	0
Antibiotic resistance genes:	
Perfect hits	0
Strict hits	2
Loose hits	0
Virulence genes	0

**Table 5:** Categorization of genes contained in the genome of *Lp. plantarum* KKP 3573 into clusters of orthologous groups.

Clusters of Orthologous Groups	*Lp. plantarum* KKP 3573	*Lp. plantarum* Pangenome
C—Energy production and conversion	3.651505	2.624688
D—Cell cycle control and mitosis	1.313261	1.633416
E—Amino acid metabolism and transport	7.078796	4.538653
F—Nucleotide metabolism and transport	4.163997	1.826683
G—Carbohydrate metabolism and transport	9.000641	6.80798
H—Coenzyme metabolism	3.042921	2.119701
I—Lipid metabolism	2.114029	1.147132
J—Translation	5.477258	1.739401
K—Transcription	9.577194	6.689526
L—Replication and repair	4.836643	17.96758
M—Cell wall/membrane/envelope biogenesis	5.605381	7.325436
N—Cell motility	0.512492	0.361596
O—Posttranslational modification, protein turnover, chaperone functions	1.825753	1.209476
P—Inorganic ion transport and metabolism	5.028828	4.033666
Q—Secondary Structure	0.832799	0.891521
T—Signal transduction	2.434337	1.677057
U—Intracellular trafficking and secretion	2.466368	1.683292
V—Defense mechanisms	1.953876	2.718204
S—Function unknown	18.57783	18.45387
No annotation	10.50609	14.55112
Total (%)	100	100

**Table 6:** *Lp. plantarum* KKP 3573 codes for genes involved in stress tolerance and survival in the beer matrix.

Locus Tag	Gene Function	Gene	E-Value
*Acid tolerance*			
MHOBIDOO_02172	Sodium proton antiporter	*yvgP*	0.0
MHOBIDOO_01677	ATP synthase subunit α	*atpA*	0.0
MHOBIDOO_01673	ATP synthase subunit a	*atpB*	4.82 × 10^−165^
MHOBIDOO_01680	ATP synthase epsilon chain	*atpC*	5.95 × 10^−74^
MHOBIDOO_01679	ATP synthase subunit β	*atpD*	0.0
MHOBIDOO_01674	ATP synthase subunit c	*atpE*	1.81 × 10-^37^
MHOBIDOO_01675	ATP synthase subunit b	*atpF*	5.41 × 10^−77^
MHOBIDOO_01678	ATP synthase γ chain	*atpG*	9.14 × 10^−213^
MHOBIDOO_01676	ATP synthase subunit delta	*atpH*	2.03 × 10^−118^
*Hop resistance*			
MHOBIDOO_02432	H(+)-stimulated, divalent metal cation uptake system	*mntH*	0.0
MHOBIDOO_00120	H(+)-stimulated, divalent metal cation uptake system	*mntH*	5.71 × 10^−301^
MHOBIDOO_01928	H(+)-stimulated, divalent metal cation uptake system	*mntH*	1.8 × 10^−290^
MHOBIDOO_00859	Unsaturated fatty acid biosynthesis	*fabZ*	8.55 × 10^−99^
MHOBIDOO_00734	Iron-dependent repressor	*mntR*	2.94 × 10^−155^
MHOBIDOO_00860	Unsaturated fatty acid biosynthesis	*fabH*	2.3 × 10^−229^
MHOBIDOO_00862	Unsaturated fatty acid biosynthesis	*fabD*	2.34 × 10^−213^
MHOBIDOO_00863	Unsaturated fatty acid biosynthesis	*fabG*	1.04 × 10^−161^
MHOBIDOO_00864	Unsaturated fatty acid biosynthesis	*fabF*	1.54 × 10^−289^
MHOBIDOO_00866	Unsaturated fatty acid biosynthesis	*fabZ2*	1.71 × 10^−91^
Bile salt tolerance:			
MHOBIDOO_03052	Linear amide C-N hydrolases, choloylglycine hydrolase family	*pva2*	4.38 × 10^−243^
MHOBIDOO_00290	Linear amide C-N hydrolase, choloylglycine hydrolase family protein	*pva1*	2.28 × 10^−250^
MHOBIDOO_00482	Linear amide C-N hydrolase, choloylglycine hydrolase family protein	*cbh*	2.83 × 10^−237^
MHOBIDOO_01519	Linear amide C-N hydrolase, choloylglycine hydrolase family protein	*yxeI*	2.83 × 10^−238^
MHOBIDOO_03052	Linear amide C-N hydrolases, choloylglycine hydrolase family	*pva2*	4.38 × 10^−243^
Extreme temperature tolerance:			
MHOBIDOO_02053	‘Cold shock’ DNA-binding domain	*cspP*	6.22 × 10^−43^
MHOBIDOO_03112	Cold shock protein	*cspA*	2.54 × 10^−42^
MHOBIDOO_00316	Cold shock protein domain	*cspL*	4.37 × 10^−43^
MHOBIDOO_00735	Cold shock protein	*cspC*	1.78 × 10^−42^
MHOBIDOO_01147	Heat shock 40 kDa protein	*dnaJ*	2.54 × 10^−266^
MHOBIDOO_01148	Heat shock 70 kDa protein	*dnaK*	0.0
MHOBIDOO_01457	Belongs to the small heat shock protein (HSP20) family	*hsp2*	2.61 × 10^−96^
MHOBIDOO_03043	Belongs to the small heat shock protein (HSP20) family	*hsp3*	4.58 ×10^−103^
MHOBIDOO_00239	Belongs to the small heat shock protein (HSP20) family	*hsp1*	2.31 × 10^−95^
MHOBIDOO_01942	Recovery of the cell from heat-induced damage, in cooperation with DnaK, DnaJ, and GrpE	*clpC*	0.0
MHOBIDOO_02252	Molecular chaperone	*GroEL*	0.0
MHOBIDOO_02253	Cochaperonin	*GroES*	1.7 × 10^−59^
MHOBIDOO_01942	Part of a stress-induced multichaperone system, it is involved in the recovery of the cell from heat-induced damage, in cooperation with DnaK, DnaJ, and GrpE	*clpC*	0.0
MHOBIDOO_02146	Belongs to the ClpA ClpB family	*clpE*	0.0
MHOBIDOO_02199	Cleaves peptides in various proteins in a process that requires ATP hydrolysis. Has chymotrypsin-like activity. Plays a major role in the degradation of misfolded proteins	*clpP*	5.11 × 10^−133^
MHOBIDOO_00438	C-terminal, D2 small domain, of ClpB protein	*clpL*	0.0
MHOBIDOO_01059	Part of a stress-induced multichaperone system, it is involved in the recovery of the cell from heat-induced damage, in cooperation with DnaK, DnaJ, and GrpE	*clpB*	0.0
MHOBIDOO_01230	ATP-dependent specificity component of the Clp protease. It directs the protease to specific substrates. Can perform chaperone functions in the absence of ClpP	*clpX*	7.8 × 10^−300^
*Osmotic shock tolerance*			
MHOBIDOO_01149	Response to hyperosmotic and heat shock	*grpE*	4.94 × 10^−115^
MHOBIDOO_00805	ABC transporter, ATP-binding protein	*opuCA*	4.51 × 10^−284^
MHOBIDOO_00806	ABC transporter permease	*opuCB*	7.11 × 10^−135^
MHOBIDOO_00807	Periplasmic glycine betaine choline-binding (lipo)protein of an ABC-type transport system (osmoprotectant binding protein)	*opuCC*	3.8 × 10^−224^
MHOBIDOO_00808	Binding-protein-dependent transport system inner membrane component	*opuCD*	1.36 × 10^−136^
Oxidative stress survival:			
MHOBIDOO_02472	Redox-regulated molecular chaperone	*hslO*	1.93 × 10^−209^
MHOBIDOO_01980	NADH dehydrogenase	*ndh*	0.0
MHOBIDOO_02214	Pyridine nucleotide–disulfide oxidoreductase, dimerization domain	*nox*	0.0
MHOBIDOO_02226	NADH oxidase	*nox*	0.0
MHOBIDOO_00557	NADH oxidase	*nox*	0.0
MHOBIDOO_01078	Pyridine nucleotide–disulfide oxidoreductase, dimerization domain	*nox*	0.0
MHOBIDOO_01095	Pyridine nucleotide–disulfide oxidoreductase, dimerization domain	*nox*	0.0
MHOBIDOO_00163	Member of the glutathione peroxidase family	*gpo*	6.07 × 10^−117^
MHOBIDOO_02589	Thiol-specific peroxidase	*tpx*	3.56 × 10^−116^
MHOBIDOO_00571	Peroxidase	*ywbN*	2.4 × 10^−230^
Biofilm formation:			
MHOBIDOO_02087	Capsular polysaccharide biosynthesis protein	*epsB*	7.88 × 10^−169^
MHOBIDOO_01363	Glycosyl transferase family 2	*epsV*	1.4 × 10^−181^
MHOBIDOO_02088	Capsular exopolysaccharide family	*ywqD*	1.43 × 10^−164^
MHOBIDOO_02836	Acetyltransferase (GNAT) domain	*ywnH*	1.66 × 10^−116^
MHOBIDOO_02208	S-ribosylhomocysteine lyase	*luxS*	2.21 × 10^−113^

## Data Availability

The *Lp. plantarum* strain KKP 3573 genome sequence has been deposited at DDBJ/ENA/GenBank under the accession JAVENX000000000.

## Supplementary Materials

The following supporting information can be downloaded at: https://www.mdpi.com/article/10.3390/genes14091710/s1, Figure S1: *Lp. plantarum* KKP 3573 carries two clusters for the production of class II bacteriocins. (A) A cluster containing genes for the production of the core peptides plantaricin E and plantaricin F. (B) A cluster containing genes for the production of the core peptides plantaricin J and plantaricin K; Table S1: Annotation of plasmid sequences in the WGS of *Lp. plantarum* KKP 3573.; Table S2: Annotation of bacteriocin synthesis clusters in the WGS of *Lp. plantarum* KKP 3573. *Lp. plantarum* KKP 3573 possesses genes for the production of plantaricins EF and JK that present sequence conservation with functionally characterized peptides produced by *Lp. plantarum* C11.
