# Supplementary material for: Depiction of the In Vitro and Genomic Basis of Resistance to Hop and High Hydrostatic Pressure of Lactiplantibacillus plantarum Isolated from Spoiled Beer
Source: Genes (Basel). 2023 Aug 28;14(9):1710. doi: 10.3390/genes14091710 (PMC10530735; doi:10.3390/genes14091710)
Supplement: Supplementary file 1 [file genes-14-01710-s001.zip › genes-2570216-supplementary/FigureS1.pdf]

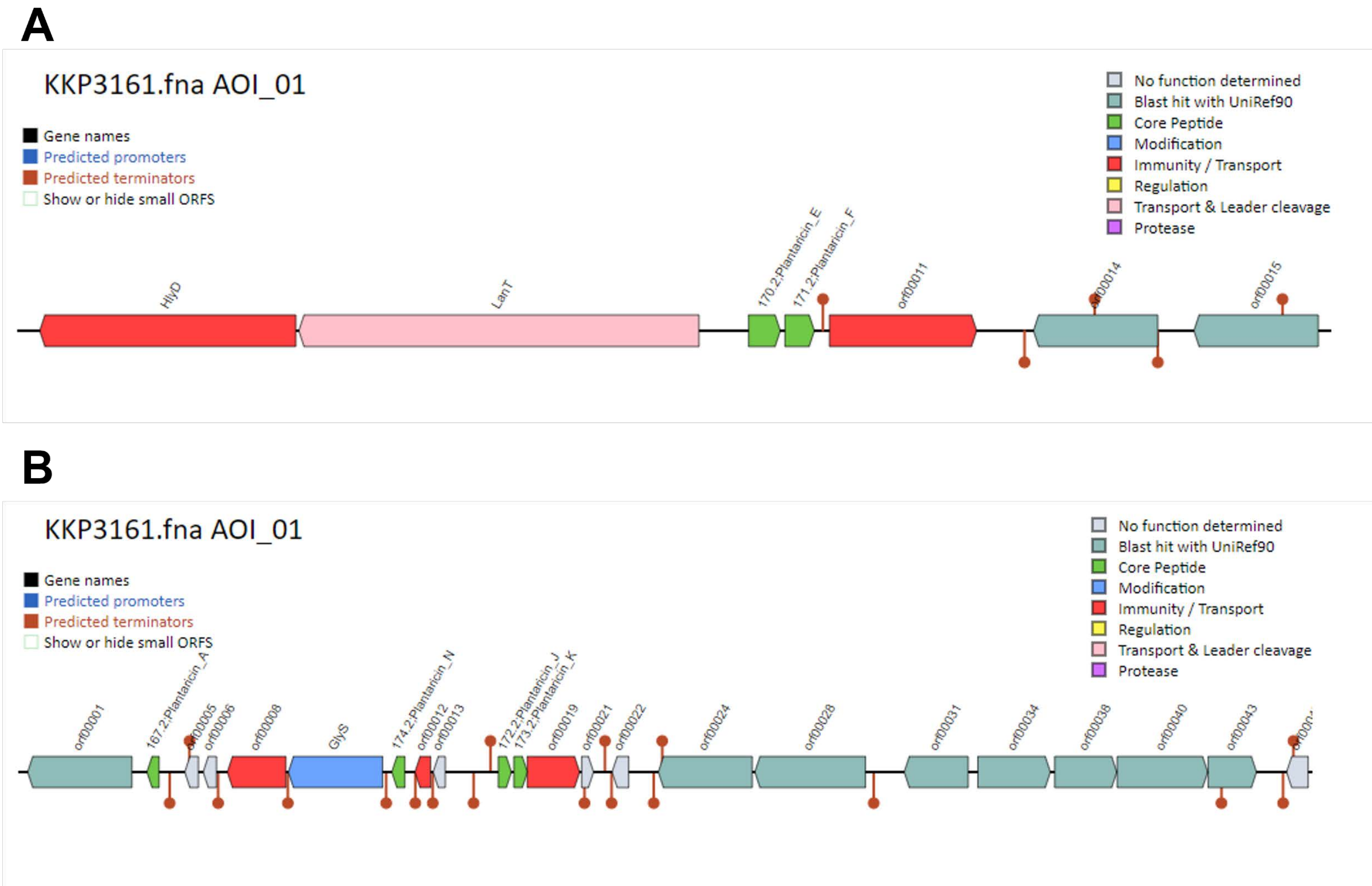
A
KKP3161.fna AOI_01
Gene names
Predicted promoters
Predicted terminators
Show or hide small ORFS
No function determined
Blast hit with UniRef90
Core Peptide
Modification
Immunity / Transport
Regulation
Transport & Leader cleavage
Protease
HlyD
LanT
170.2;Plantaricin_E
171.2;Plantaricin_F
orf00011
orf00014
orf00015
B
KKP3161.fna AOI_01
Gene names
Predicted promoters
Predicted terminators
Show or hide small ORFS
No function determined
Blast hit with UniRef90
Core Peptide
Modification
Immunity / Transport
Regulation
Transport & Leader cleavage
Protease
orf00001
167.2;Plantaricin_A
orf00005
orf00006
orf00008
GlyS
174.2;Plantaricin_N
orf00012
orf00013
172.2;Plantaricin_J
173.2;Plantaricin_K
orf00019
orf00021
orf00022
orf00024
orf00028
orf00031
orf00034
orf00038
orf00040
orf00043
